# Challenges in diagnosing and managing multifocal atrial tachycardia

**DOI:** 10.1016/j.hrcr.2022.12.017

**Published:** 2023-02-15

**Authors:** Margaret Harvey

**Affiliations:** Department of Acute and Tertiary Care, College of Nursing, University of Tennessee Health Science Center, Memphis, Tennessee

**Keywords:** Multifocal atrial tachycardia, Atrial fibrillation, Supraventricular tachycardia, Palpitation, Chronic obstructive pulmonary disease, 12-Lead electrocardiogram

## Introduction

Multifocal atrial tachycardia (MAT) can be difficult to correctly diagnose and is often misinterpreted as atrial fibrillation. This case report highlights the importance of accurate diagnosis, as management vastly differs and has significant implications.

## Case report

**Figure 1:**
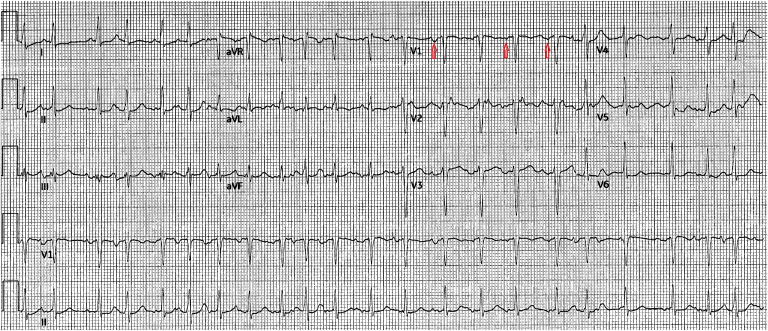
Twelve-lead electrocardiogram of multifocal atrial tachycardia with P waves most visible in V_1_.

The patient is a 69-year-old woman admitted to the hospital for shortness of breath and hypoxemia secondary to chronic obstructive pulmonary disease (COPD) exacerbation with a history of COPD, cardiomyopathy, coronary artery disease, and hypertension. She was referred to the cardiac electrophysiology team for irregular tachycardia and reported intermittent palpitations for several months, with baseline shortness of breath at rest that worsens with exertion, and denies dizziness, syncope, chest pain, orthopnea, or paroxysmal nocturnal dyspnea. Pertinent home medications include metoprolol succinate, losartan, atorvastatin, clopidogrel, Xopenex, and Symbicort. The baseline 12-lead electrocardiogram (ECG) upon admission revealed sinus tachycardia at 111 beats per minute. The following day, the patient had multiple ECGs done for irregular tachycardia that were mistakenly analyzed by the machine algorithm as an undetermined rhythm and atrial fibrillation with rapid ventricular response. A review of the ECG demonstrated multiple morphologies of P waves consistent with MAT (Figure 1). An echocardiogram demonstrated left ventricular ejection fraction 36%–40%, mild right ventricular enlargement, grade II diastolic function, mild left atrial enlargement, and moderate tricuspid regurgitation. A nuclear medicine stress test showed a small fixed defect and a partially reversible defect. A computed tomography scan of the chest showed cardiomegaly, pulmonary arterial hypertension, pulmonary edema, and emphysema. The patient received guideline-directed medical therapy for COPD, coronary artery disease, and congestive heart failure, resulting in improvements in overall condition. The electrophysiology team added low-dose digoxin and increased the metoprolol dose for rate control with resolution of MAT and restoration to sinus rhythm by discharge.

## Discussion

Owing to the irregular rhythm observed in MAT, confirming diagnosis with baseline and subsequent 12-lead ECGs is essential, as the rhythm can easily be confused with atrial fibrillation based on physical examination and a single ECG tracing. MAT is defined as an irregular, rapid rhythm with 3 distinct P waves of different morphology identified on the ECG.^1^ Unlike atrial fibrillation, there is a distinct isoelectric period with varying P-P, P-R, and R-R intervals.^1^ The mechanism of action of MAT is not well understood but is associated with certain conditions, such as pulmonary, coronary, and valvular disease. Management can be challenging and includes treating the underlying disease, slowing conduction at the atrioventricular (AV) node level, and administering supplemental magnesium. Antiarrhythmic agents and cardioversion have been shown to be ineffective. Rate control is achieved with a beta-blocker or calcium channel blockers when no contraindications are present (class of recommendation IIa).^1^ More recently, metoprolol has been found to be superior to verapamil and should be used with caution in patients with decompensated pulmonary disease, sinus node dysfunction, or AV block.^2^ For patients who are refractory to drug therapy with left ventricle dysfunction due to recurrent MAT, AV nodal ablation followed by pacing (preferable biventricular or His bundle pacing) should be considered.^2^ Finally, anticoagulation is not indicated for patients with MAT because they are not at risk for stroke, unlike patients with atrial fibrillation.^1^

## Conclusion

This case report highlights the importance of accurate rhythm diagnosis for MAT, as it may be misinterpreted as atrial fibrillation with profound management implications.
